# Screening of potential hub genes involved in Kidney Wilms tumor via bioinformatics analysis and experimental validation

**DOI:** 10.1186/s12885-024-12541-x

**Published:** 2024-06-27

**Authors:** Qiang Zeng, Tingting Liu, Lilu Qin, Chen Wang, Guangbei Peng, Zhong Liu, Junfeng Tao

**Affiliations:** 1 Department of Pediatric Surgery, Jiangxi Maternal and Child Health Hospital, Nanchang, 330100 Jiangxi China; 2 Department of Pediatric Surgery, Jiangxi Hospital Affiliated to Children’s Hospital of Chongqing Medical University, Nanchang, 330100 Jiangxi China

**Keywords:** Wilms tumor, Molecular biomarkers, EMCN, CCNA1

## Abstract

**Background:**

Wilms tumor (WT) is the most common pediatric embryonal tumor. Improving patient outcomes requires advances in understanding and targeting the multiple genes and cellular control pathways, but its pathogenesis is currently not well-researched. We aimed to identify the potential molecular biological mechanism of WT and develop new prognostic markers and molecular targets by comparing gene expression profiles of Wilms tumors and fetal normal kidneys.

**Methods:**

Differential gene expression analysis was performed on Wilms tumor transcriptomic data from the GEO and TARGET databases. For biological functional analysis, Gene Ontology (GO) and Kyoto Encyclopedia of Genes and Genomes (KEGG) pathway enrichment were utilized. Out of 24 hub genes identified, nine were found to be prognostic-related through univariate Cox regression analysis. These nine genes underwent LASSO regression analysis to enhance the predictive capability of the model. The key hub genes were validated in the GSE73209 datasets, and cell function experiments were conducted to identify the genes’ functions in WiT-49 cells.

**Results:**

The enrichment analysis revealed that DEGs were significantly involved in the regulation of angiogenesis and regulation of cell differentiation. 24 DEGs were identified through PPI networks and the MCODE algorithm, and 9 of 24 genes were related to WT patients’ prognosis. EMCN and CCNA1 were identified as key hub genes, and related to the progression of WT. Functionally, over-expression of EMCN and CCNA1 knockdown inhibited cell viability, proliferation, migration, and invasion of Wilms tumor cells.

**Conclusions:**

EMCN and CCNA1 were identified as key prognostic markers in Wilms tumor, suggesting their potential as therapeutic targets. Differential gene expression and enrichment analyses indicate significant roles in angiogenesis and cell differentiation.

**Supplementary Information:**

The online version contains supplementary material available at 10.1186/s12885-024-12541-x.

## Introduction

Wilms tumor (WT) is the most common pediatric renal malignancy [1]. The outcomes for patients with Wilms’ tumors (WTs) have significantly improved over the last decades, because of the advances in multi-model therapy including surgery, chemotherapy, and radiation therapy, which allowed most patients with > 90% overall survival for those with localized, and 80% for those with metastatic nonanaplastic WT [2–4]. Surgical removal of the diseased kidney has limitations because current treatments are not entirely appropriate for some populations, especially infants and children, and patients with bilateral tumors [5, 6]. Therefore, the key to improving patient prognosis is to improve treatment based on clinical and biological risk factors, and further stratification of current treatment options based on the prognostic value of tumor biology would be an important approach to improving WT prognosis.

In previous studies, Wilms tumor was associated with various genetic changes, identification of which yields diagnostic, prognostic, and therapeutic advances, including cancer genes driver mutations, epigenetic remodellers, microRNA processing genes, and the transcription factors [7–10]. Further understanding of the genetic basis and differentially expressed genes (DEGs) are of great value in identifying disease biomarkers and pathogenesis, which leads to identifying novel therapeutic targets and advances toward personalized medicine. At present, there is no reliable biomarker for Wilms tumor and the molecular mechanism of its occurrence and development have not been fully clarified.

In this study, we aimed to identify potential prognostic and therapeutic targets, and potential molecular biological mechanisms through TARGET and GEO database. Targeting therapeutic targets with prognostic significance, as well as the molecular biological mechanism, may provide promising therapeutic avenues for patients with this disease.

## Methods

### Data collection and preprocessing

**Fig. 1:**
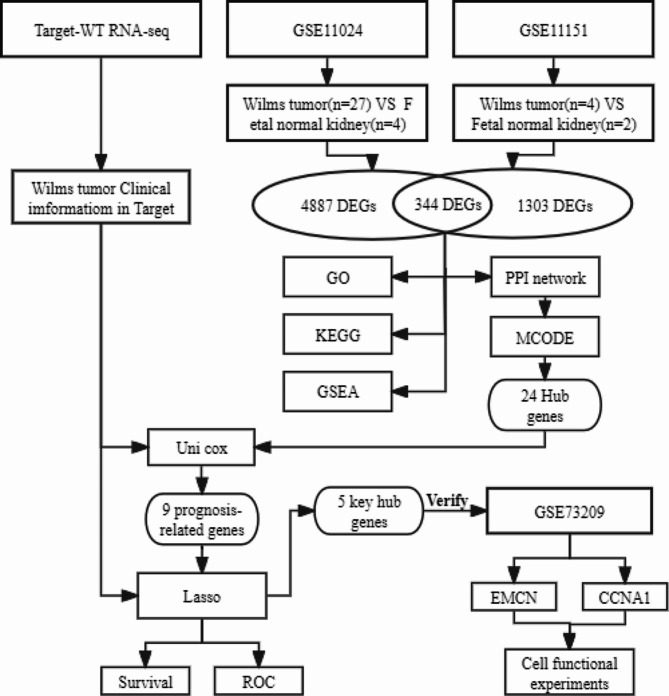
Flow chart of the analysis process in our study

The workflow of the study was represented in Fig. 1. A large amount of RNA-seq data and clinical information were obtained from the Therapeutically Applicable Research to Generate Effective Treatments (TARGET) database. We downloaded the GSE11151, GSE11024 and GSE73209 datasets from the NCBI GEO database (http://www.ncbi.nih.gov/geo). The functions of the datasets in our manuscript were shown in Table 1. In each GEO dataset, we only extracted the samples of the Wilms tumor and fetal normal kidney samples for subsequent analysis.

**Table 1:** Characteristics of the included datasets

Dataset	Country	Platforms	No. of samples	Usage here
GSE11151	Netherlands	GPL570	Wilms tumor(*n* = 4), fetal normal kidney (*n* = 2)	Identification of hub genes
GSE11024	USA	GPL6671	Wilms tumor(*n* = 27), fetal normal kidney(*n* = 4)	Identification of hub genes
TARGET-WT	USA	RNA seq	DAWT(*n* = 22), FHWT(*n* = 114)	Construction and identification of the prognostic model
GSE73209	Sweden	GPL10558	Wilms tumor(*n* = 32), fetal normal kidney(*n* = 4)	Verification of hub genes

### Differential gene expression analysis

The differentially expressed genes between Wilms tumor(WT), and fetal normal kidney tissue from the GEO database were downloaded. The raw data were downloaded as MINiML files. Using the limma package in the R software to study the differentially expressed genes. The adjusted P-value was analyzed to correct the false positive results in GEO datasets. Adjusted *P* < 0.05 and Log (Fold Change) > 1 or Log (Fold Change) < − 1 were defined as differentially expressed genes (DEGs). Then, we compared the DEGs identified from GSE11151 with those DEGs from the GSE11024 using a Venn diagram and calculated the overlap coefficient between the two gene sets. The dataset GSE73209 was used to verify the hub genes.

### GO and KEGG pathway enrichment analysis

The GO enrichment analysis and KEGG pathway enrichment analysis were used to identify the biological functions and pathways associated with the intersection of differentially expressed genes (DEGs) derived from TARGET and GEO data. The cluster profile package was used for GO enrichment analysis and KEGG pathway enrichment analysis, adjusted p-values for multiple testing using the Benjamini-Hochberg method, and less than 0.05 as significantly enriched. The BP, MF, and CC categories separately and applied a filter of a minimum count of 10 genes per GO term. GO biological processes gene sets, GO cellular components gene sets, and GO molecular functions gene sets were obtained from the Molecular Signatures Database (MSigDB) as reference gene sets [11]. The R package enrich plot was conducted to visualize the enriched GO terms and KEGG pathways using dot plots and bar plots [12].

### PPI network analysis

A PPI network between DEGs was constructed based on the Search Tool for the Retrieval of Interacting Genes/Proteins (STRING) database (http://string-db.org) with a confidence level ≥ 0:400 [13]. Then, the PPI file was imported into Cytoscape 3.9.1 (http://cytoscape.org/) to visualize and analyze the PPI network [14]. The hub genes were screened with the MCODE algorithm using default settings.

### Survival analysis

Survival analysis was used to evaluate the prognostic value of the key hub genes in WT. The median expression value of each gene or module eigengene was used as the cutoff to divide the patients into high and low-expression groups. The relationship between the mRNA expression levels of hub genes and the prognosis (OS/PFS) was analyzed through the Kaplan-Meier analysis. The genes or modules with log-rank p-values less than 0.05 were considered as significantly associated with survival. The hazard ratio (HR) and 95% confidence interval (CI) for each gene were calculated by using the log-rank tests and univariate Cox proportional hazards regression.

### Construction and identification of the prognostic risk model

To confirm the potential prognosis-related hub genes. The expression matrix integrating the initial genes of the model with patient survival status and survival time was constructed. The LASSO regression algorithm was used for feature selection, and 10-fold cross-validation was used to determine the parameters among which the key genes associated with the patient survival cycle were screened. We calculated the risk score of each patient based on the regression coefficient of the hub genes in the signature and the corresponding expression value of the hub genes. The risk score was calculated using the following formula:

$$\eqalign{{\rm{ Risk score }} & & = & {\rm{ expression of Gene }}1*\alpha 1 \cr + {\rm{ expression of Gene }}2*\alpha 2 \cr + \ldots {\rm{ expression of Genen }}*\alpha {\rm{, }} \cr}$$

Risk score = expression of Gene1∗α1 + expression of Gene2∗α2+…expression of Genen∗αn,

where α represents the regression coefficient of the hub genes in the signature. Based on the median risk score, the patients were divided into high-risk and low-risk groups. Kaplan–Meier survival curve analysis was carried out to compare the OS and PFS between the high-risk group and the low-risk group. A p-value < 0.05 was selected as the significant cutoff value. The time ROC (v 0.4) analysis was used to compare the predictive accuracy of the risk score.

### Cell culture

The HEK 293T cells and WiT49 cell line were donated by Dr. Tanpeng Chen. It is a Wilms’ tumor (WT) cell line that is derived from the first-generation xenograft of a human WT lung metastasis. Some differentiation potential is retained by WiT49 cells, displaying the so-called “triphasic” histology when grown in tissue culture plates [15–17]. The HEK 293T cells were cultured in DMEM (HyClone) with 10% FBS (Gibco), 2 mmol/L L-glutamine (Sigma), 100 U/mL penicillin, and 100 µg/mL streptomycin (Gibco). WiT49 cells were cultured in DMEM with 15% FBS (Gibco), 2 mmol/L L-glutamine (Sigma), 100 U/mL penicillin, 100 µg/mL streptomycin (Gibco), 0.5 mL/L 2-mercaptoethanol (Invitrogen), and 6 mg/L insulin. All cell lines were proven to be mycoplasma negative.

### Transfection

Transfection was conducted using Lipofectamine 2000 (Invitrogen, USA) following the manufacturer’s instructions. DNA plasmid (2.5 µg) or siRNA (100 nM) was diluted in 250 µL Opti-MEM (Gibco) and incubated for 5 min.Lipofectamine 2000 (5 µL) was diluted in 250 µL Opti-MEM and incubated for 5 min, then combined with the DNA or siRNA and incubated for 20 min. The complexes were added to cells in 1.5 mL fresh DMEM without antibiotics and incubated at 37 °C for 4–6 h. The medium was replaced with DMEM containing antibiotics and FBS, and cells were incubated for 24–72 h. Transfection efficiency was evaluated using fluorescence microscopy for GFP-expressing plasmids 24 h post-transfection. Quantitative PCR (qPCR) was conducted to measure gene expression levels 48 h post-transfection, and protein levels were assessed by Western blotting 72 h post-transfection. Controls included non-transfected cells and cells transfected with non-targeting siRNA or empty vector. All experiments were performed in triplicate. Si RNA-CCNA1, si-NC, EMCN mimic, and mimic-NC were obtained from Rubibio Company (Guangzhou, China). The CCNA1 targeting siRNA, negative control (NC) siRNA, EMCN mimic, and OE-NC sequences were showed in Table 2.

**Table 2:** Oligo sequences

Gene	Target sequence
Negative Control (si-NC)	TTCTCCGAACGTGTCACGT
Si RNA-CCNA1-1	GTGTTATTCTGGATCAGAAAATG
Si RNA-CCNA1-2	GACATCTACATGGATGAACTAGA
EMCN mimic	EMCN-F: ATGGAACTGCTTCAAGTGACCATT
	EMCN-R: TCAGTTCTTGGTTTTTCCTTGTGCAG

### Western blotting

Total proteins were extracted from WiT49 and HEK 293T. The protein concentration was detected with the BCA protein assay. Then, 25 µg total protein samples were subjected to SDS-PAGE, and the separated bands were transferred to 0.22 μm PVDF membranes. Protein was blocked for 1 h with a blocking solution. The membrane was incubated with the primary antibody overnight at 4 °C and with the secondary antibody at room temperature for 1 h. Finally, the gel was imaged. Anti-EMCN (1:1000 dilution, PA5-21395, Thermo Fisher Scientific), Anti-CCNA1(1:1000 dilution, 13295-1-AP, Proteintech) and anti-α-Tubulin (1:5000 dilution, 11224-1-AP, Proteintech). α-Tubulin was used as a loading control.

### RT-PCR

Cellular RNA was extracted from WT cells (WiT-49) and normal renal epithelial cells (293T). We then synthesized cDNA from the total RNA samples using an M-MLV reverse transcription kit (M1705, Promega). Quantitative PCR was performed on the resulting cDNA samples using the SYBR Master Mix (DRR041B, TAKARA). The expression of CCNB1 and GAPDH was based on the formula 2^^-ΔΔCt^. Table 3 lists the primers that were utilized.

**Table 3:** Primers used for quantitative real time PCR.

Oligonucleotides	Sequence (5′–3′)
GAPDH-F	GTGGGCAAGGTATCCTG
GAPDH-R	GATTCAGTGTGGTGGGGGAC
EMCN-F	TGCAGGACTTTCTCCTTTTC
EMCN-R	ATTTGTTCTGGTGGGTTTGT
CCNA1-F	GCACACTCAAGTCAGACCTGCA
CCNA1-R	ATCACATCTGTGCCAAGACTGGA

### Cell viability and plate clone formation assay

Transfected WiT49 was seeded in 96-well plates at a density of 3,500 cells/well for 24 h. Ten microliters of Cell Counting Kit-8 (CCK-8, Dojindo, Japan) reagent was added into each well at 24, 48, and 72 h posttransfection. After incubating at 37 °C and 5% CO_2_ for 2 h, the absorbance (450 nm) was recorded using a plate reader (Pulang New Technology, Beijing, China).

For the plate clone formation assay, 600 cells per well were seeded in a 6-well plate and cultured for 12 days. The culture medium was changed every 4 days. Then, the cells were fixed in 4% formaldehyde and stained with crystal violet. Cell clones were counted and analyzed.

### Transwell assay

Transfected WiT49 was resuspended in a serum-free medium and seeded to the top chamber (Corning, USA) with or without precoating of Matrigel (BD Bioscience, USA). A complete medium with 15% FBS was added to the bottom chamber. After culturing for 24 h at 37 °C and 5% CO2, the invaded or migrated cells at the bottom side of the transwell membrane were fixed with 4% paraformaldehyde for 10 min and stained with 0.1% crystal violet (Solarbio, China) at room temperature for 30 min. After washing 3 times with phosphate-buffered saline (PBS), the number of migrated cells was counted under a phrase contrast microscope (Nikon, Japan).

### Statistical analysis

R software (version 4.0.3) for data analysis and visualization. The Kruskal–Wallis test was used for continuous variable data, and the wilcoxon test was used to compare the differences between the two groups. The risk ratio (HR) and 95% confidence interval (CI) were estimated using the survival package of the Cox regression model. Kaplan-Meier method and log-rank test to compare the survival curves between different groups. Two-tailed P values to determine the statistical significance of the differences, and considered them significant when *P* < 0.05 (*P < 0.05, **P < 0.01, ***P < 0.001).

## Result

### Identification of the differential expressed genes in WT patients

**Fig. 2:**
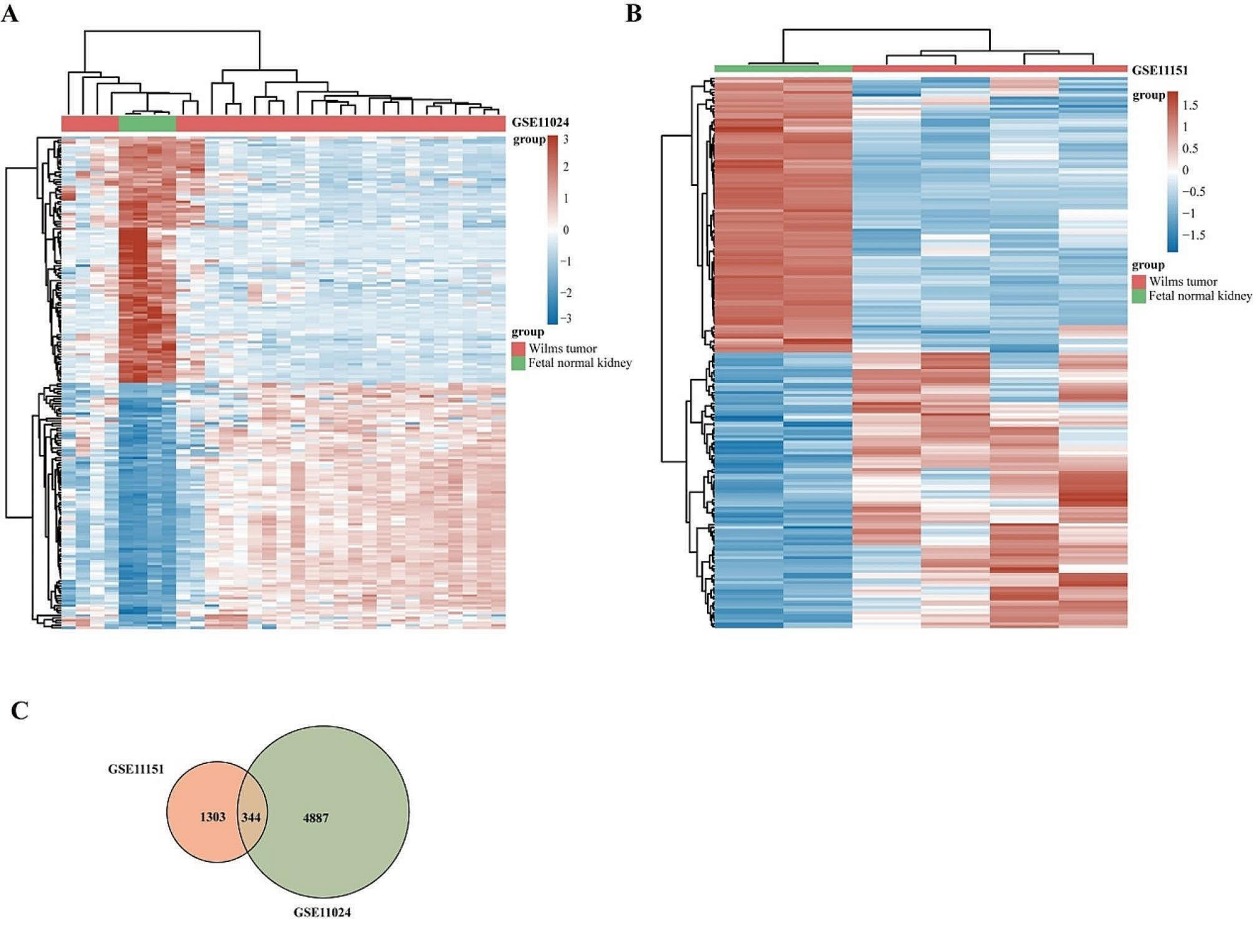
Differentially expressed genes. (**A**) Heatmap plots of DEGs in GSE11024. (**B**) Heatmap plots of DEGs in GSE11151. (**C**) plot showing intersecting genes. Green circle represents DEGs of GSE11024 and red circle represents DEGs of GSE11151

The gene expression data were obtained from the GEO database. In the GSE11024 dataset, compared with fetal normal kidney samples (*n* = 4), we obtained 2954 up-regulated and 2674 down-regulated DEGs in WT samples (*n* = 27) (Fig. 2A). Comparing WT samples (*n* = 4) and fetal normal kidney samples (*n* = 2) in the GSE11151 dataset, 1647 DEGs were identified and then screened 548 up-regulated genes and 1099 down-regulated genes (Fig. 2B). After deleting the duplicates and micro-RNA, 344 potential marker targets were obtained and displayed with Venn map (Fig. 2C).

### Functional enrichment analyses

**Fig. 3:**
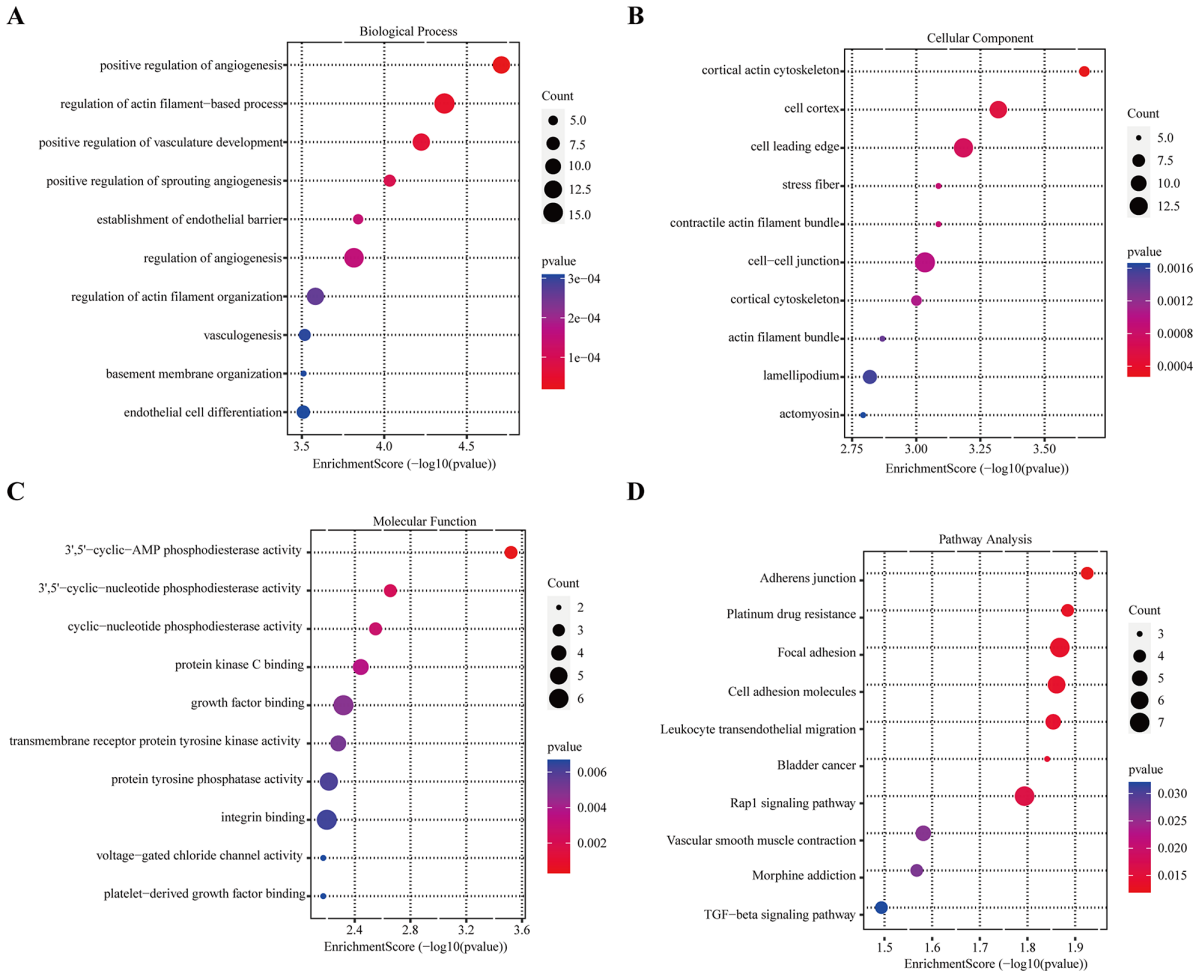
Functional enrichment analysis of the DEGs. (**A**) GO enrichment of DEGs in biological process terms. (**B**) GO enrichment of DEGs in cellular component terms. (**C**) GO enrichment of DEGs in molecular function terms. (**D**) Enriched KEGG pathways of the DEGs

To further investigate the potential mechanisms and biological functions of the DEGs, we conducted GO enrichment analysis, and KEGG pathway enrichment on the 344 differentially expressed genes. The function enrichment analysis yielded 402 GO terms, including 311 Biological Process terms (BP). These terms primarily encompassed positive regulation of angiogenesis, regulation of actin filament-based process, and positive regulation of vasculature development (Fig. 3A). Additionally, 45 Cellular Component terms (CC) were identified, such as cortical actin cytoskeleton, cell cortex, and cell leading edge (Fig. 3B). Furthermore, 46 terms of Molecular Function (MF) were obtained, with notable categories being protein kinase C binding and growth factor binding (Fig. 3C). In the KEGG pathway analysis, the differentially expressed genes were predominantly associated with adherens junction, platinum drug resistance, and focal adhesion (Fig. 3D).

### Identification and verification of hub genes

**Fig. 4:**
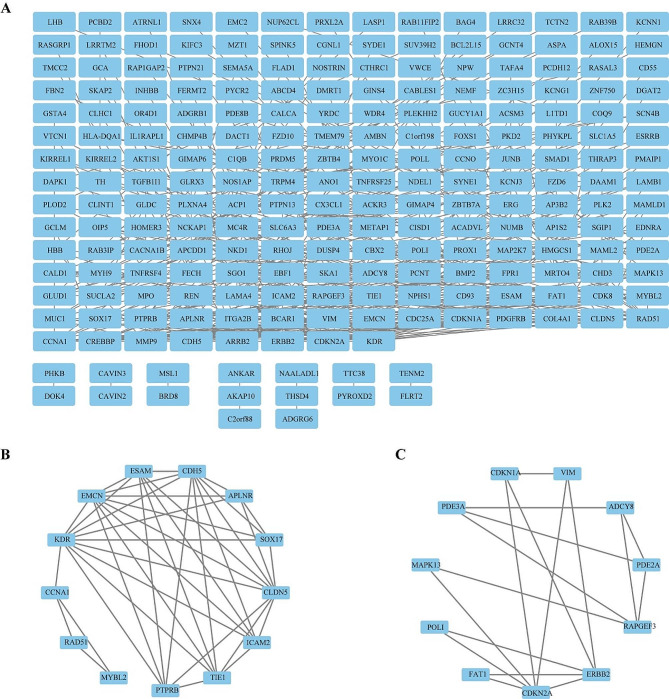
Construction of the PPI network. (**A**) PPI network of the DEGs. (**B**) The hub genes of intersected PPI network in cluster 1. (**C**) The hub genes of intersected PPI network in cluster 2

To identify the hub genes in WT, we imported the 344 terms of DEGs into the STRING database to construct the PPI network. The network contains a total of 220 nodes (genes) and 424 edges, with 124 genes not directly related to other targets (Fig. 4A). We then exported the data to construct the network in Cytoscape 3.9.1. By calculating the top 2 cluster network through MCODE, we found a total of 24 hub genes in the top 2 cluster network. Cluster 1 includes 13 hub genes: ICAM2, KDR, MYBL2, CLDN5, RAD51, SOX17, PTPRB, EMCN, CCNA1, CDH5, TIE1, ESAM, and APLNR (score = 6.667, 13 nodes and 40 edges) (Fig. 4B). Cluster 2 includes 11 hub genes: RAPGEF3, FAT1, VIM, POLI, PDE3A, PDE2A, MAPK13, CDKN2A, ADCY8, ERBB2, CDKN1A (score = 3.6, 11 nodes and 18 edges) (Fig. 4C).

### Analysis and validation of key hub genes prognostic in patients with Wilms tumor

**Fig. 5:**
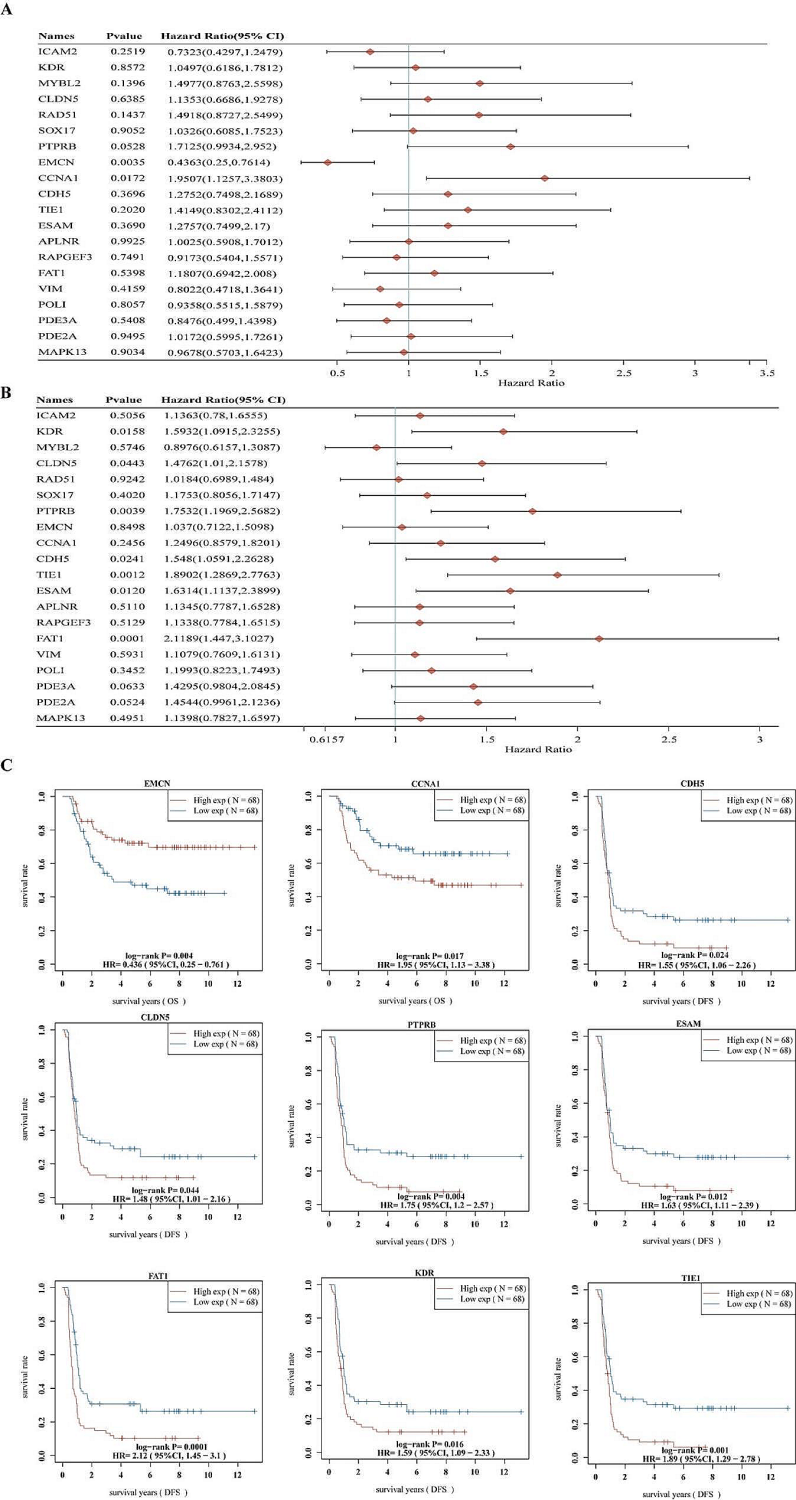
Identification of prognostic genes through Cox univariate analysis. (**A**) Univariate Cox regression analysis of prognostic factors for overall survival. (**B**) Univariate Cox regression analysis of prognostic factors for disease free survival. (**C**) Overall survival (OS) and disease-free survival analysis of prognostic genes in the TARGET-WT database

To assess the independent prognostic ability of the 24 hub genes, we conducted a univariate Cox regression analysis to compare the survival differences among these groups with differential gene expression. The forest plot in Fig. 5A&B displays the prognostic relevance of the 24 hub genes. Subsequently, we identified nine genes that exhibited correlation with the progression of WT patients. In the analysis of overall survival, low EMCN expression was associated with a poor overall survival rate (*p* = 0.004, HR = 0.436). Patients with high expression of CCNA1 had significantly worse overall survival compared to those with low CCNA1 levels (*p* = 0.017, HR = 1.95). KM plotting analysis indicated that seven genes with high expression were linked to unfavorable disease-free survival in WT patients, namely CDH5 (*p* = 0.024, HR = 1.55), CLDN5 (*p* = 0.044, HR = 1.48), ESAM (*p* = 0.012, HR = 1.63), FAT1 (*p* = 0.0001, HR = 2.12), KDR (*p* = 0.016, HR = 1.59), PTPRB (*p* = 0.004, HR = 1.75), and TIE1 (*p* = 0.001, HR = 1.89) (Fig. 5C).

### Construction of the prognostic risk model and analysis

To further analyze the predictive ability of 9 prognostic-related genes, we constructed two prognostic risk modes based on the above 9 hub genes to predict the overall survival and disease-free survival of WT patients.

**Fig. 6:**
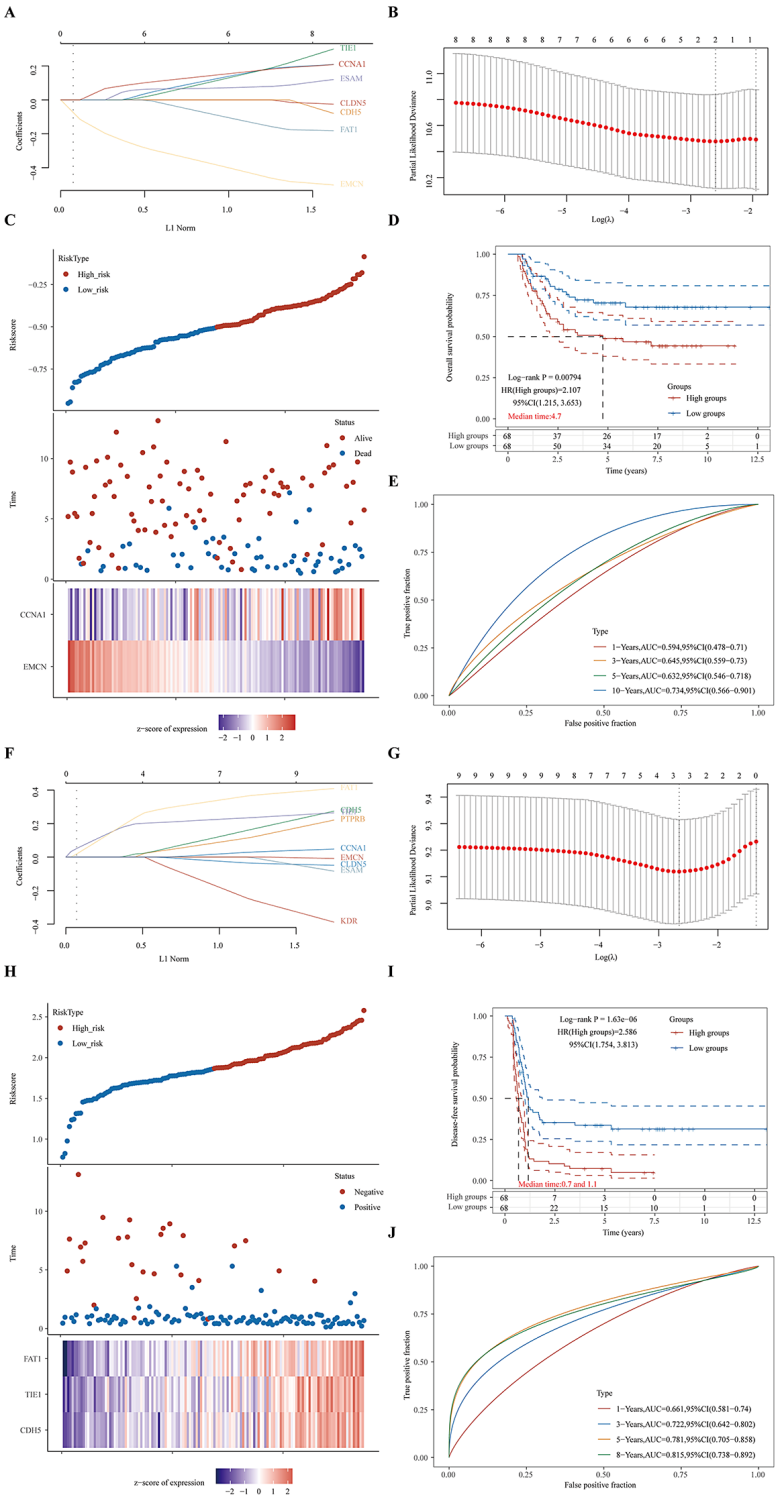
Prognostic risk score model analysis of 9 prognostic genes in WT patients. (**A**)&(**F**) The coefficients of selected features are shown by lambda parameter. (**B**)&(**G**) The partial likelihood deviance versus log (λ) was drawn using the LASSO Cox regression model. (**C**)&(**H**) The patients ranked by risk score, corresponding survival status and heatmap of the risk model. (**D**)&(**I**) The Kaplan–Meier survival analysis of the gene signatures according to the median cutoff value. (**E**)&(**J**) The time-dependent ROC analysis of the gene signature

Nine genes underwent LASSO regression analysis to enhance the predictive capability of the model (Fig. 6A&B). Ultimately, two hub genes displayed significant associations with patients’ overall survival. A risk-score formula was developed: Riskscore=(-0.1614)*EMCN+ (0.0378)*CCNA1 (lambda.min = 0.0743). These findings indicated that CCNA1 was a risk factor, while EMCN was a protective factor in the overall survival risk model. Additionally, a prediction model based on the two gene signatures was constructed, and Fig. 6C presents the distribution of the risk score, survival status, and corresponding heatmap of the expression level of the two hub genes in patients. Using the median risk score as the threshold, the sample was divided into high-risk and low-risk groups. Notably, the overall survival of WT patients in the low-risk group surpassed that of patients in the high-risk group (*p* = 0.00794, HR = 2.107) (Fig. 6D). To further assess the accuracy of the prognostic risk model in predicting the hub genes of WT patients, time-dependent ROC curves were analyzed. The respective areas under the curve (AUCs) of the prognostic signature reached 0.594, 0.645, 0.632, and 0.734 at 1, 3, 5, and 10 years, respectively (Fig. 6E).

Similarly, we identified three hub genes significantly associated with patients’ disease-free survival (Fig. 6F&G&H). The risk score was calculated using the following equation: Riskscore = (0.0128) * CDH5 + (0.1921) * TIE1 + (0.2201) * FAT1 (lambda.min = 0.0703). CDH5, TIE1, and FAT1 were considered risk factors in the disease-free survival risk model. The disease-free survival of WT patients in the low-risk group was better than that of patients in the high-risk group (*p* < 0.001, HR = 2.586) (Fig. 6I). The respective areas under the curve (AUCs) of the prognostic signature reached 0.661, 0.722, 0.781, and 0.815 at 1, 3, 5, and 8 years, respectively (Fig. 6J).

### Analysis and validation of key hub genes expression in WT patients

**Fig. 7:**
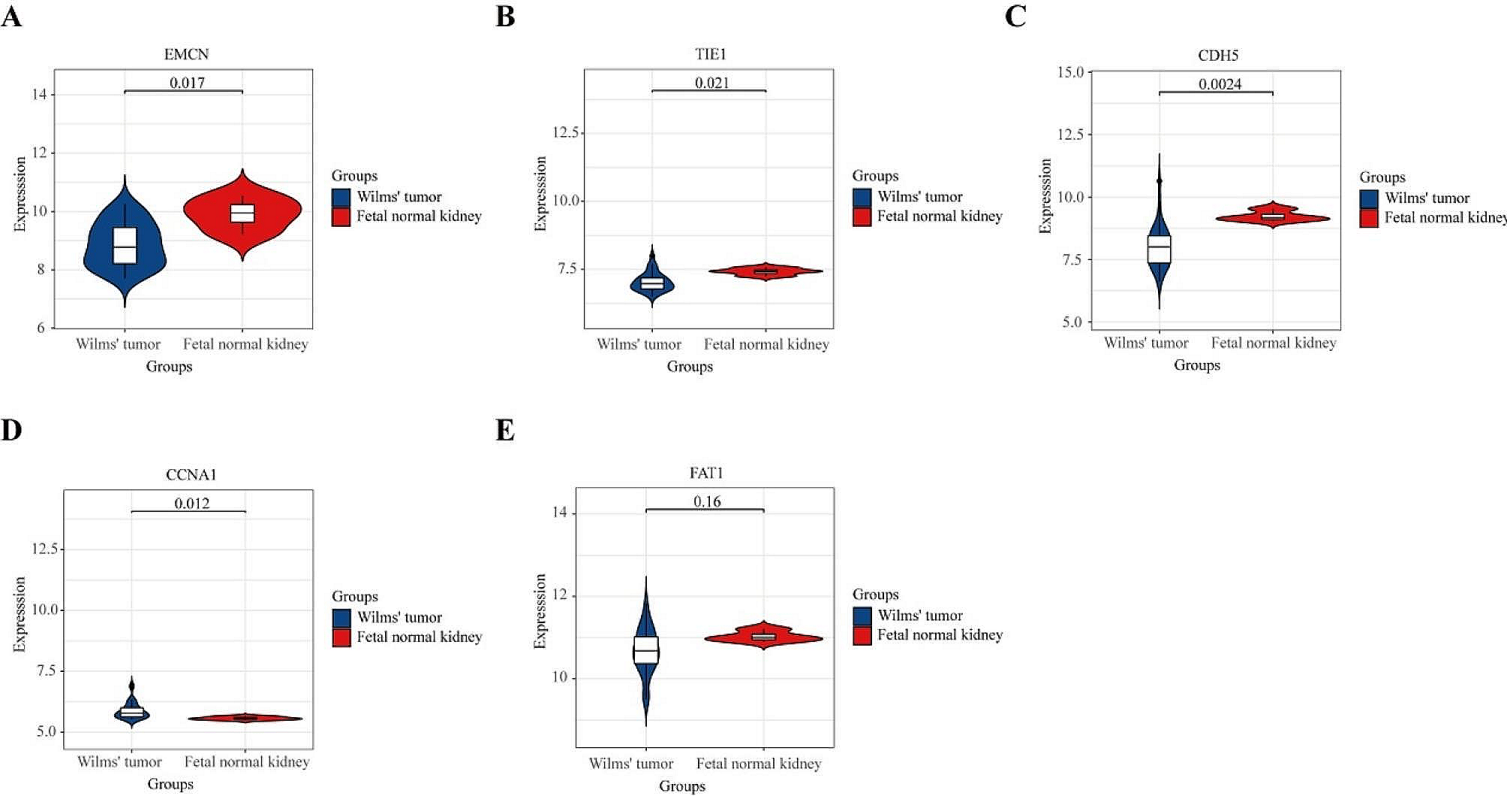
Validation of hub genes performance in external datasets. (**A**)EMCN gene expression. (**B**) TIE1 gene expression. (**C**) CDH5 gene expression. (**D**) CCNA1 gene expression. (**E**) FAT1 gene expression

To validate the expression of genes in WT patients, we utilized GEO database (GSE73209) to analyze the 5 main hub gene expressions in Wilms’ tumor and Fetal normal kidney. In the external validation cohort, EMCN (Fig. 7A), TIE1 (Fig. 7B), and CDH5 (Fig. 7C) were significantly upregulated in fetal normal kidney tissues. Conversely, CCNA1 (Fig. 7D) was significantly overexpressed in Wilms tumor samples. No significant expression differences were observed for FAT1 (Fig. 7E). Ultimately, only the outcomes validated by EMCN and CCNA1 were consistent with the prognostic risk model. Consequently, we proceeded to utilize these two genes for subsequent in vitro functional assessments.

#### Cell functional verification

**Fig. 8:**
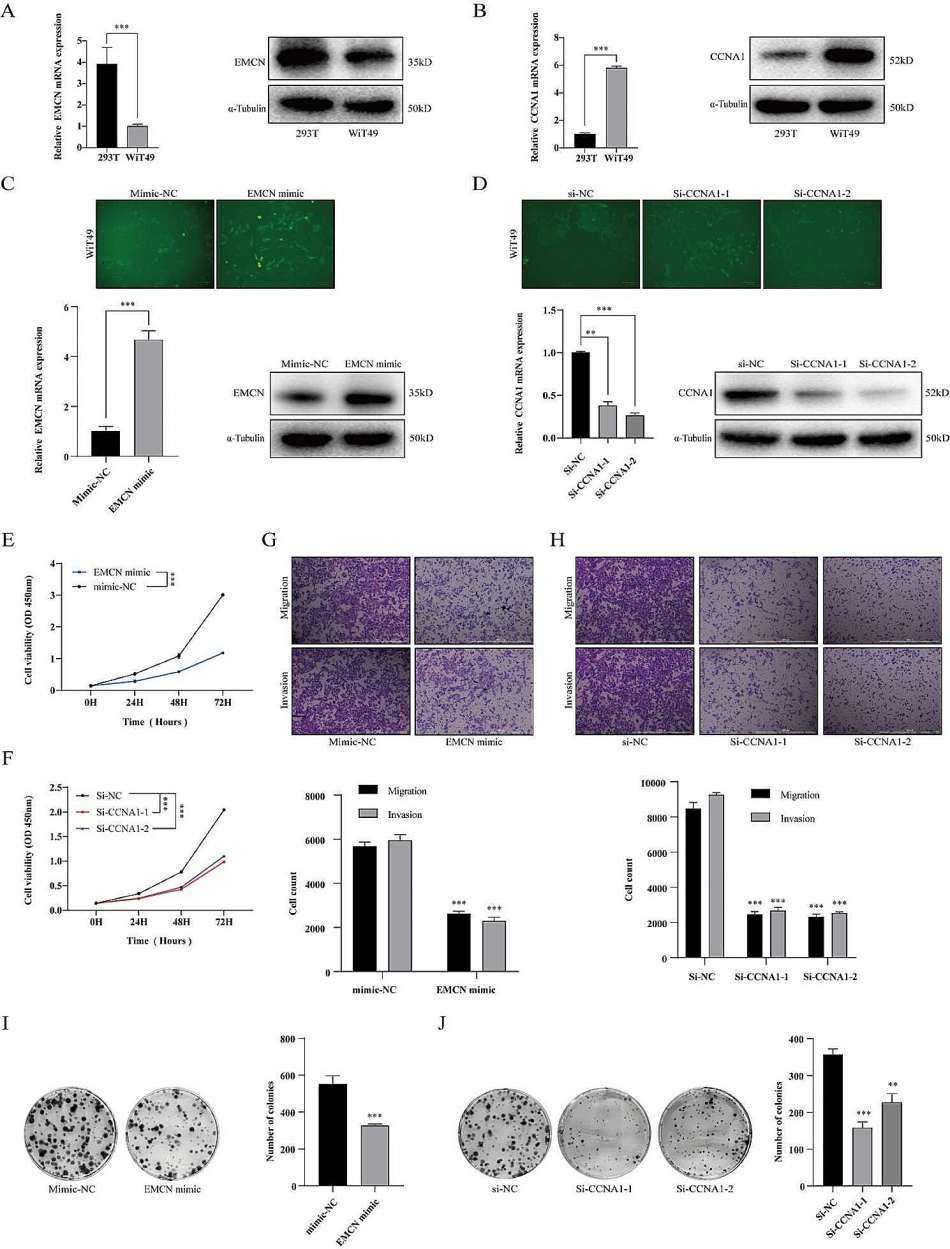
Functional validation of EMCN and CCNA1 in WiT-49 cells. Expression analysis of the EMCN gene in the cell line. Expression analysis of the CCNA1 gene in the cell line. WiT-49 cells were transfected with EMCN mimic or mimic NC or (**D**) WiT-49 cells were transfected with CCNA1 siRNA or siNC. Forty-eight hours after transfection, green fluorescent protein (GFP) fluorescence was visualized to monitor transfection efficiency. Relative mRNA and protein expression of EMCN or CCNA1 were detected by RT-qPCR and Western blot. (**E**) CCK8 were used to determine the cell viability of WiT-49 cells transfected with EMCN mimic or mimic NC and (**F**) WiT-49 cells transfected with CCNA1 siRNA or siNC. (**G**) Transwell assay were used to determine the cell migration and invasion of WiT-49 cells transfected with EMCN mimic or mimic NC and (H) WiT-49 cells transfected with CCNA1 siRNA or siNC. (**I**) Colony-forming assays were used to determine the cell proliferation of WiT-49 cells transfected with EMCN mimic or mimic NC and (**J**) WiT-49 cells transfected with CCNA1 siRNA or siNC.

The EMCN gene was downregulated in RNA and protein in WT cells (Fig. 8A), while the CCNA1 gene was upregulated in RNA and protein in WT cells (Fig. 8B). To investigate the roles of EMCN and CCNA1 in WT cells, we conducted transfection experiments to validate their functions. EMCN mimic, mimic-NC, SiRNA-CCNA1, and si-NC were separately introduced into WIT-49 cells. After 48 h of transfection, we assessed the overexpression efficiency of the EMCN mimic and the knockdown efficiency of si-CCNA1 (Fig. 8C&D). Additionally, functional studies on cells revealed that overexpressing EMCN and silencing CCNA1 significantly reduced the cell viability, proliferation, migration, and invasive capacity of WiT-49 cells (Fig. 8E-J).

## Discussion

Wilms tumor (WT) is the second most common intra-abdominal tumor and the most common primary renal tumor in children [6]. It accounts for about 5% of malignancies in children under 15 years old [18]. Approximately 75% of children with WT develop the condition between the ages of 1 and 5 years [19]. The dimension of accessible experimental features is limited due to a lack of typical Wilms tumor cell lines [20]. Therefore, the identification of potential molecular biological mechanisms and differential expressed genes (DEGs) with prognostic significance specific to WT tissue is a promising approach for better understanding this disease.

In the present study, we performed differential gene expression analysis on WT transcriptomic data from the GEO databases. All data were normalized before being analyzed into DEGs. Using the “limma” package in R language, we identified 5628 DEGs from the GSE11024 and 1647 DEGs from the GSE11151 matrix file. By using the Venn map production website, we discovered 344 potential marker targets that were DEGs between Wilms tumor tissue and fetal normal kidney samples. Previous studies have analyzed Wilms tumor using the GEO database. For example, Avčin SL et al. [21]. identified 43 miRNAs that are differentially expressed in Wilms tumor regardless of histological type. Huang et al. [22]. found 25 key genes associated with WT prognosis and developed a prediction model using 12 gene signatures. However, there are few articles in Wilms tumor research that specifically use fetal normal kidney samples as normal controls and consider different histology classification subgroups for analysis. Therefore, our study has notable strengths.

The Gene Ontology (GO) function, and Kyoto Encyclopedia of Genes and Genomes (KEGG) pathway were utilized for the biological functional analysis after obtaining the 344 DEGs by analyzing the key DEGs. Our observations in Wilms tumor suggested that regulation of angiogenesis and regulation of cell differentiation play a key role in WT. Angiogenesis is a prerequisite for growth and metastasis of solid tumors. The experimental human WT displays a vascular architecture that is driven by vascular endothelial growth factor [23, 24], and anti-angiogenesis can lead to WT growth inhibition and also decrease the incidence and size of metastases [25–28]. All WTs consist of variable proportions of blastemal, epithelial, and stromal components in histology. Stromal and epithelial components may show varying degrees and lines of differentiation, including heterologous elements [29]. In the SIOP-RTSG 2016 Wilms tumor pathology and molecular biology protocol, histologic subtyping is based on the assessment of percentages of chemotherapy-induced changes and viable tumor components [30]. It has been shown that preoperative chemotherapy (PCT) can induce further differentiation and maturation, which also confirms the regulation of cell differentiation plays a key role in WT [31]. Therefore, our functional enrichment analysis results suggest these signaling pathways may serve a crucial role in Wilms tumor progression.

PPI network was established by STRING database analysis, and 2 modules that might serve an important role in the development of Wilms tumor which contains 24 hub genes were detected by further using the MCODE algorithm in Cytoscape software. The result indicates that EMCN was identified as a protective factor, while another four genes (CCNA1, CDH5, TIE1, FAT1) were identified as risk factors. Finally, we used the external validation set (GSE73209) to further verify the expression of the above 5 genes and obtained 2 hub genes with expression consistent with the aforementioned analysis. The central role of EMCN and CCNA1 within this network highlights their potential as biomarkers for prognosis and targets for therapeutic intervention. The involvement of these genes in critical biological processes such as cell proliferation, apoptosis, and angiogenesis underscores their importance in WT pathogenesis. The regulation of angiogenesis and cell differentiation aligns with the roles of EMCN and CCNA1, respectively.

Endomucin (EMCN) is a transmembrane O-sialylated protein expressed on the surface of the endothelium and can affect tube morphogenesis of endothelial cells in vitro and leukocyte adhesion to endothelial cells in the blood [32–34]. EMCN has been suggested as a prognostic signature of gastric cancer [35], and the loss of EMCN has been identified can drive tumor lung metastasis through the premetastatic niche [36]. EMCN was down-regulated in ccRCC tissues, compared with normal kidney tissues, and overall survival was decreased in EMCN lowly expressed ccRCC patients [37]. Our findings suggest that EMCN downregulation in WT is associated with poor prognosis, which aligns with its known role in promoting endothelial cell stability and inhibiting metastasis. The loss of EMCN may disrupt vascular integrity and enhance metastatic potential by facilitating a pre-metastatic niche. This mechanism is supported by previous studies indicating that EMCN loss can drive tumor lung metastasis through the pre-metastatic niche. In WT, reduced EMCN expression could impair the vascular architecture within the tumor microenvironment, promoting angiogenesis and tumor progression.

Cyclin A1 (CCNA1) is an important cell cycle regulator in the G1/S phase [38, 39]. CCNA1 was identified as a downstream player in p53-dependent apoptosis and G2 arrest [40]. DNA methylation status of CDKNA2 and CCNA1 were correlated with treatment response to doxorubicin and 5-fluorouracil in locally advanced breast tumors, which might be the cause of acquired drug resistance in breast cancer [41]. Hypermethylation of CCNA1 has been correlated to breast cancer progression [42]. Huang KC et al. observed that the overexpression of CCNA1 was associated with cellular resistance to the antineoplastic agent paclitaxel [43]. Overexpression of CCNA1 in WT patients was found to be correlated with poor prognosis, suggesting its role in uncontrolled cell proliferation. In WT, CCNA1 overexpression may facilitate cell cycle progression and proliferation, while its role in apoptosis resistance could enhance tumor survival and growth. This indicates that EMCN and CCNA1 may play an important role in Wilms tumor development. Through cell function experiments, we found that over-expression EMCN and silencing CCNA1 gene greatly reduced WiT-49 cell viability, proliferation, migration, and invasive capacity. The identification of EMCN and CCNA1 as key regulators in WT provides a rationale for exploring targeted therapies that modulate their expression. Strategies to enhance EMCN expression or inhibit CCNA1 could potentially improve clinical outcomes. Furthermore, understanding the downstream pathways influenced by these genes may reveal additional targets for combination therapies.

In summary, this study elucidates the mechanisms by which EMCN and CCNA1 contribute to WT progression, highlighting their roles in angiogenesis, cell cycle regulation, and response to chemotherapy. These insights pave the way for developing targeted therapies aimed at improving prognosis and treatment efficacy for WT patients. Future research should focus on validating these findings in larger cohorts and exploring the therapeutic potential of modulating EMCN and CCNA1 expression. However, this study has several limitations. Firstly, the prognostic analysis and model development were based on retrospective data from public cohorts, introducing potential bias due to unbalanced clinicopathological features and treatment heterogeneity. Secondly, Wilms tumor (WT) is a relatively rare primary malignancy, and the sample size in this study was limited, necessitating further validation in larger, independent cohorts to ensure the robustness and generalizability of the findings. Thirdly, while the in vitro assays validated the roles of EMCN and CCNA1 in WT progression, the underlying downstream mechanisms of these genes remain unclear and require further in-depth in vivo studies to elucidate their functional pathways and interactions. Moreover, the study did not explore the potential impact of genetic and epigenetic alterations on the expression and function of the identified hub genes, which could provide additional insights into WT pathogenesis. Future studies should incorporate advanced machine-learning algorithms and comprehensive bioinformatics approaches to refine the prognostic models and explore additional molecular targets. Collaborative efforts to collect larger clinical samples and integrate multi-omics data will enhance the understanding of WT and improve the clinical applicability of the findings. These improvements will contribute to more accurate prognostic predictions and the development of targeted therapeutic strategies for WT.

## Conclusions

We identified EMCN and CCNA1 as prognostic signatures associated with the progression of WT by performing a series of bioinformatics analyses and cell function experiments. The results of our study will be of great importance in elucidating the potential molecular biological mechanism of Wilms tumors and developing new prognostic markers and molecular targets.

### Electronic supplementary material

Below is the link to the electronic supplementary material.

Supplementary Material 1

Supplementary Material 2

## Data Availability

The datasets used and/or analyzed during the current study are available from the corresponding author upon reasonable request. The data sources and software tools used in this study are detailed in the Methods section of the manuscript.

## Abbreviations

WT: Wilms tumors
TARGET: Therapeutically Applicable Research to Generate Effective Treatments database
GEO: Gene Expression Synthesis database
STRING: Search Tool for the Retrieval of Interacting Genes/Proteins
MSigDB: Molecular Signatures Database
DEGs: Differentially expressed genes
GO: Gene Ontology
BP: Biological Process terms
CC: Cellular Component terms
MF: Molecular Function
KEGG: Kyoto Encyclopedia of Genes and Genomes
PPI: protein-protein interaction
CCNA1: Cyclin A1
EMCN: Endomucin
ATCC: American Type Culture Collection
RT-qPCR: reverse transcription-quantitative polymerase chain reaction
GAPDH: glyceraldehyde 3-phosphate dehydrogenase
CCK-8: Cell Counting Kit-8
NC: Normal control
