# Supplementary material for: Screening of potential hub genes involved in Kidney Wilms tumor via bioinformatics analysis and experimental validation
Source: BMC Cancer. 2024 Jun 27;24:771. doi: 10.1186/s12885-024-12541-x (PMC11209955; doi:10.1186/s12885-024-12541-x)
Supplement: Supplementary file 1 — Supplementary Material 1 [file 12885_2024_12541_MOESM1_ESM.docx]

Fig 9A Expression analysis of the EMCN gene in the cell line.

EMCN-35kd
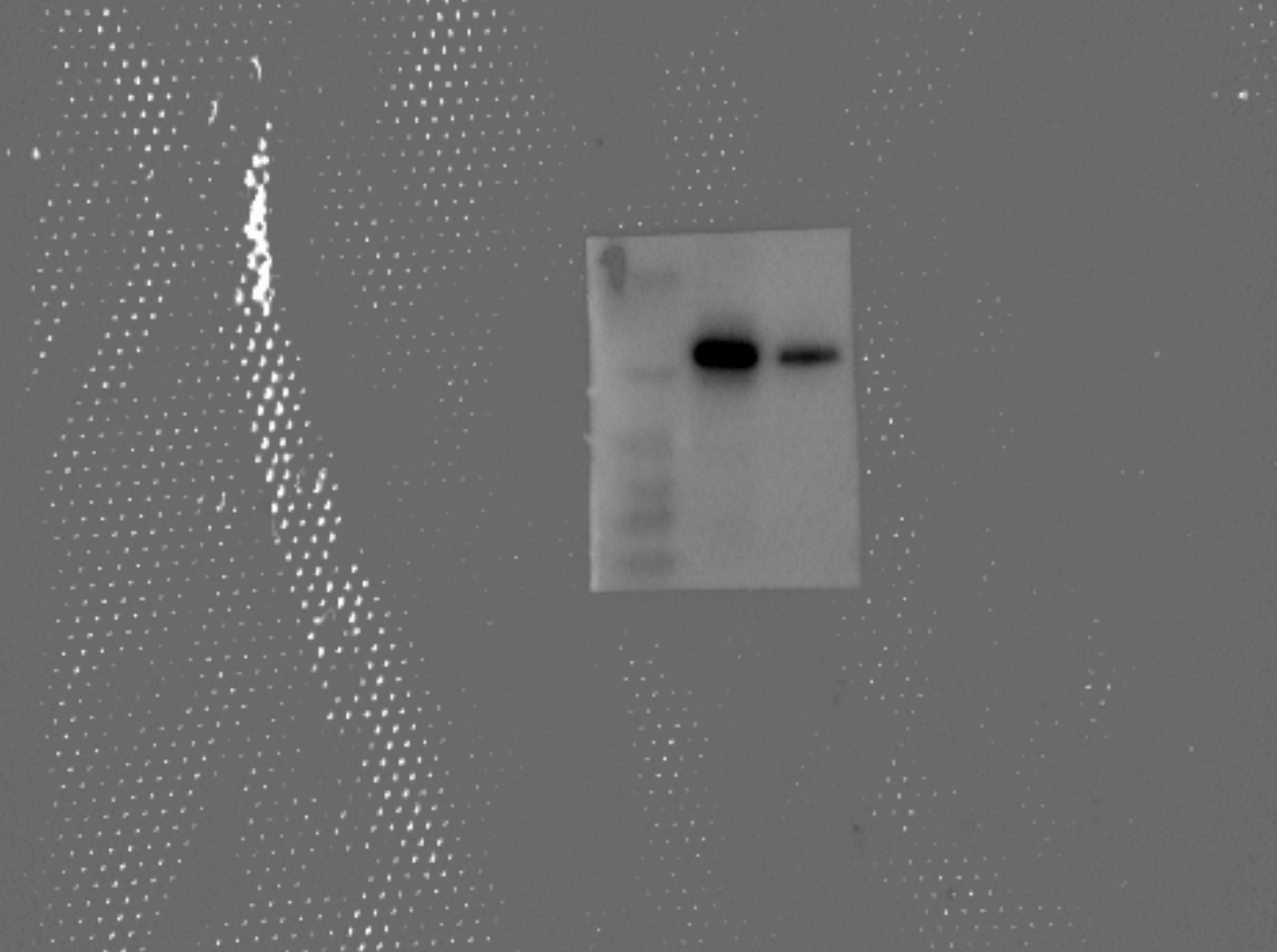


293T

WiT-49

Tublin-50kd
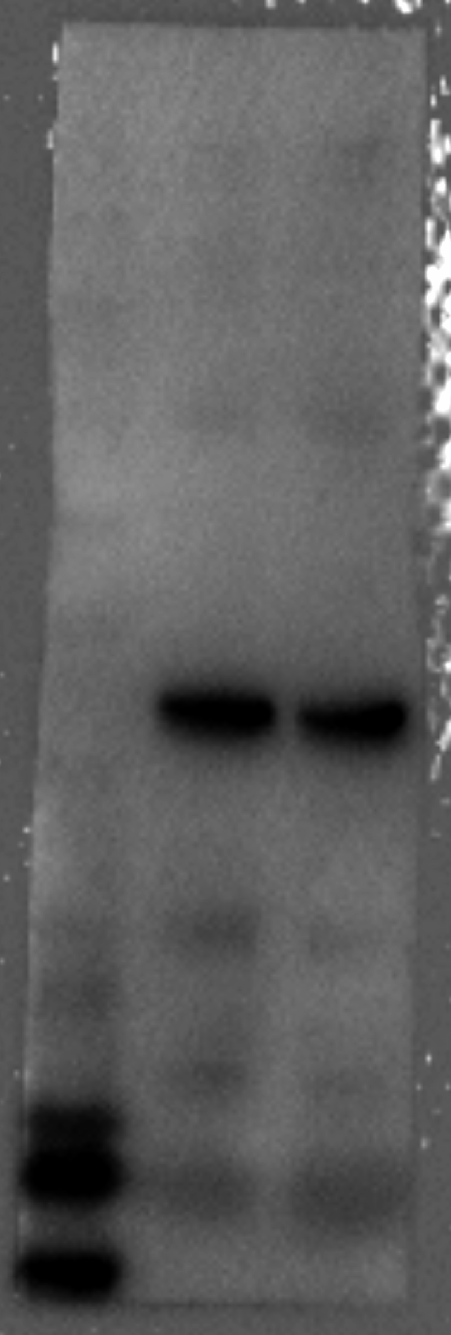


WiT-49

293T

Fig 9B Expression analysis of the CCNA1 gene in the cell line.

CCNA1-52kd
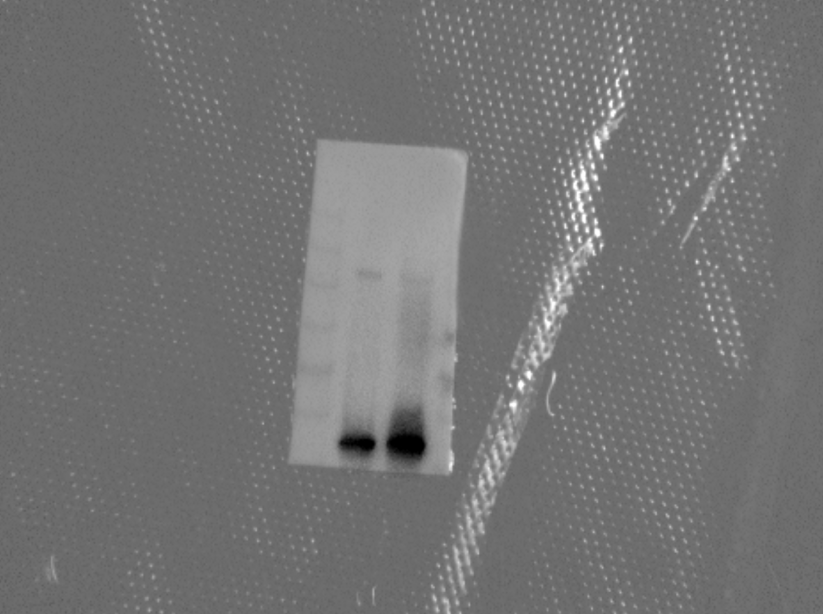


WiT-49

293T

Tublin-50kd
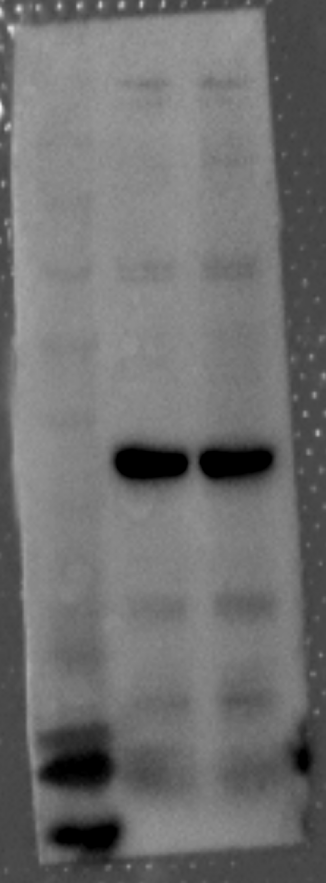


WiT-49

293T

Fig 9C WiT-49 cells were transfected with EMCN mimic or mimic NC

EMCN-35kd
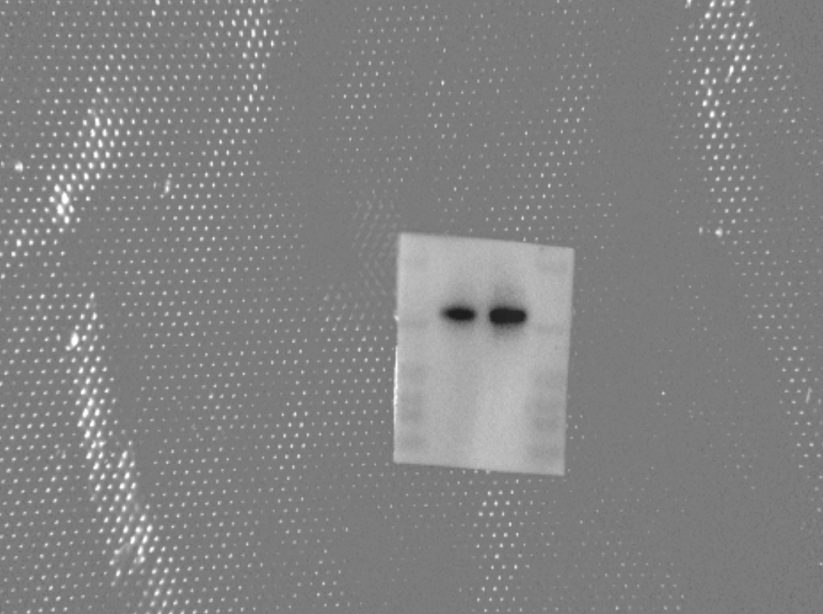


mimic NC

EMCN mimic

Tublin-50kd
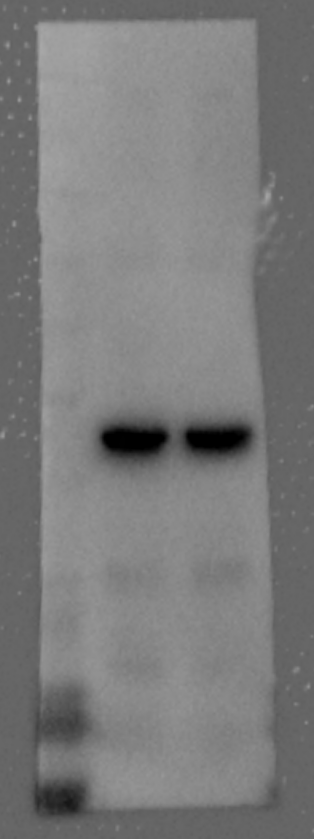


mimic NC

EMCN mimic

Fig 9D WiT-49 cells were transfected with CCNA1 siRNA or siNC.

CCNA1-52kd
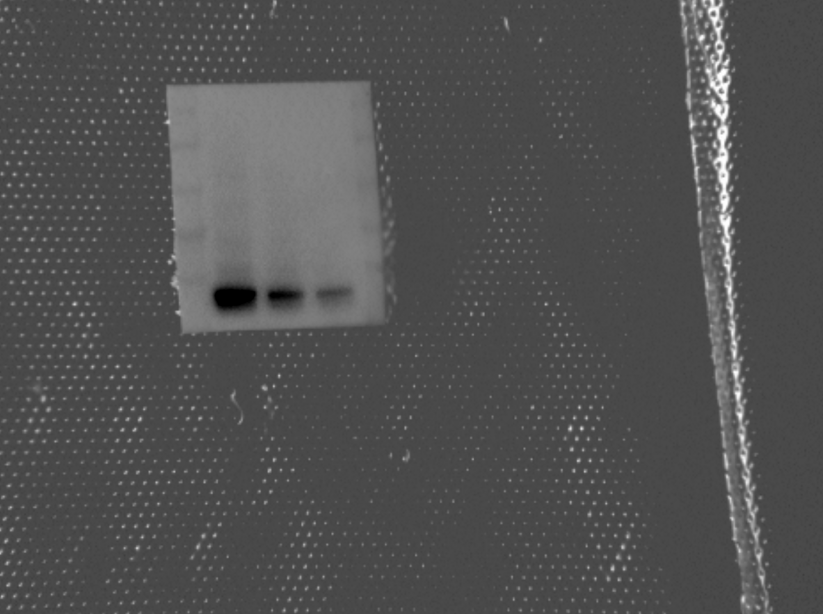


Si NC

Si CCNA1-1

Si CCNA1-2

Tublin-50kd
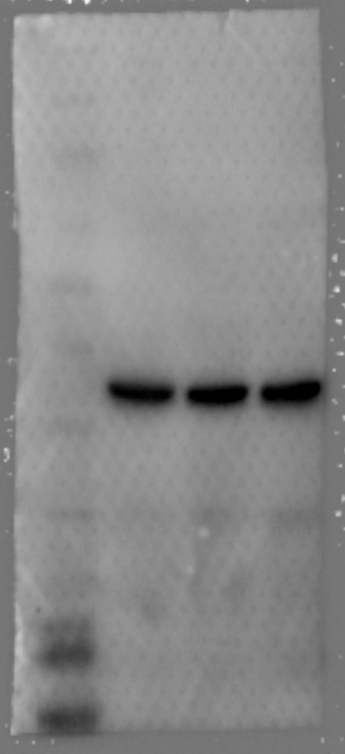


Si CCNA1-2

Si NC

Si CCNA1-1
